# Supplementary material for: Dendritic cell-targeted therapy expands CD8 T cell responses to bona-fide neoantigens in lung tumors
Source: Nat Commun. 2024 Mar 13;15:2280. doi: 10.1038/s41467-024-46685-y (PMC10937682; doi:10.1038/s41467-024-46685-y)
Supplement: Supplementary file 1 — Supplementary Information [file 41467_2024_46685_MOESM1_ESM.pdf]

SUPPLEMENTARY INFORMATION

Dendritic cell-targeted therapy expands CD8 T cell responses to *bona-fide* neoantigens in lung tumors.

Content

This file contains Supplementary Fig. 1 to 6, Supplementary Tables 1-3, and the uncropped western blot membrane.

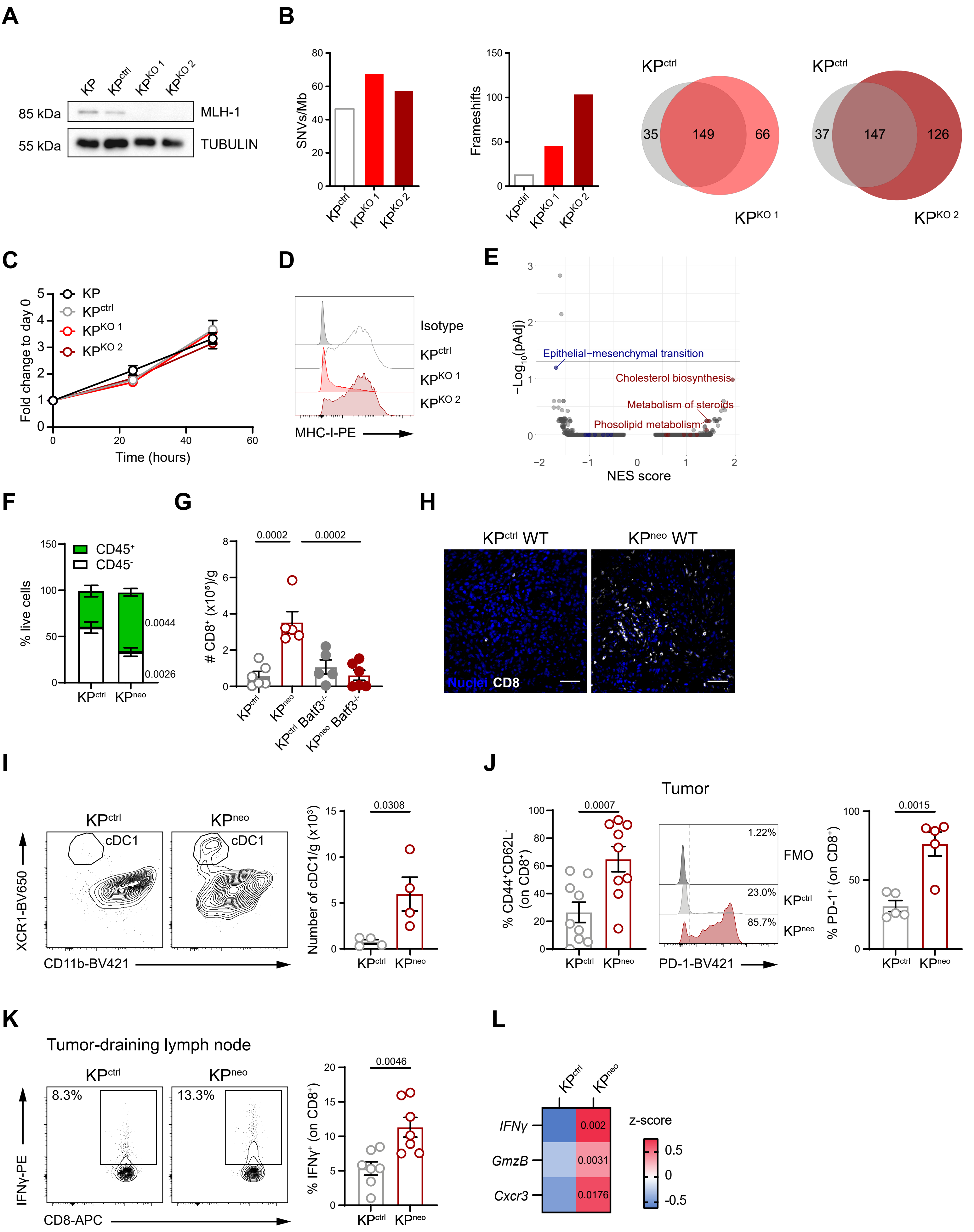

Supplementary Fig. 1

Supplementary Fig. 1| Generation and characterization of KP^{neo} cells.

A) *Mlh1* was inactivated by CRISPR-CAS9 transient transfection. KP^{ctrl} cells were generated by transient CAS9 transfection without targeting vector. Cells were subcloned after transfection and screened for MLH1 expression by Western Blot. One representative membrane out of four independents performed. **B)** KP^{ctrl} cells and two *Mlh1*-deficient clones (KP^{KO1}, KP^{KO2}) were sequenced (whole exome sequencing) to determine the tumor mutational load. Bars show single nucleotide variants (SNVs/Mb) (left) and frameshifts (right) in each clone. The Venn diagram depicts shared and unique predicted neoAgs calculated using NetMHCI 4.0 package and the mouse reference C57 genome as a baseline. **C)** Growth of the parental KP line and KP^{ctrl}, KP^{KO1} and KP^{KO2} was evaluated *in vitro* at 3 time points. Data refers to numbers of cells and are plotted as fold change over day 0. n=3 technical replicates from one representative experiment out of three performed. **D)** Levels of MHC class-I were assessed by flow cytometry (FC) after IFNγ stimulation. Representative histograms from one independent experiment out of three performed. **E)** Gene set enrichment on RNAseq data from KP^{neo} and KP^{ctrl} cells (n=3), showed no changes in pathways related to cell proliferation, metabolism, inflammatory responses and antigen processing (using Reactome and Hallmarks databases). NES (Normalized Enrichment Score), KP^{neo} vs KP^{ctrl} adjusted p values (adj. p-value). **F-K)** KP^{ctrl} and KP^{neo} cells were implanted s.c. in wild type and Batf3 deficient animals. Tissues were harvested at day 21 to analyze the immune infiltrate by FC and visualize by confocal microscopy. **F)** Bars show cell fractions of CD45^{+} and CD45^{-} cells among live cells in tumor masses (n=9, two polled experiments). **G)** Absolute numbers of CD8 T cells infiltrating tumors in wild-type and Batf3$^{-/-}$ hosts (n=6 for KP^{ctrl} and KP^{neo} Batf3$^{-/-}$, n=6 for KP^{neo} and KP^{ctrl} Batf3$^{-/-}$, one out of two independent experiments). **H)** Representative tissue cryosections showing localization of CD8 T cells within tumor nodules. One representative image from one out of four animals per group, from one independent experiment out of three performed. Scale bars represent 50 μm. **I)** of cDC1 infiltrating the tumor mass at day 21 post-tumor challenge (n=4, one out of two independent experiments). **J)** Expression of effector/effector memory markers (CD44^{+}/CD62L^{-}) and PD-1 on tumor infiltrating CD8 T cells (left n=9, two pooled experiments; right n=5, one out of two independent experiments). **K)** Tumor- draining lymph node cells (tdLN) were stimulated *ex-vivo* with PMA/Ionomycin to determine the fraction of IFNγ^{+} CD8^{+} T cells by intracellular staining (ICS). Flow cytometry analysis and quantification (n=7, pooled data form two experiments). **L)** Relative expression of the indicated genes (RT-qPCR) in KP^{ctrl} and KP^{neo} tumors. Heat map showing the z-score of the relative expression (RT-qPCR) of the indicated genes in total tumor tissues (n=7, pooled data from two experiments). Two-way ANOVA followed by Tukey's post-test in **C**, **G**; or Sidak's post-test in **F;** and two-tailed *t-test* in **I,J**; and two-tailed Multiple *t-test* with Holm-Sidak correction method in **K**. All data are plotted as mean ± SEM. Source data are provided as a Source Data File.

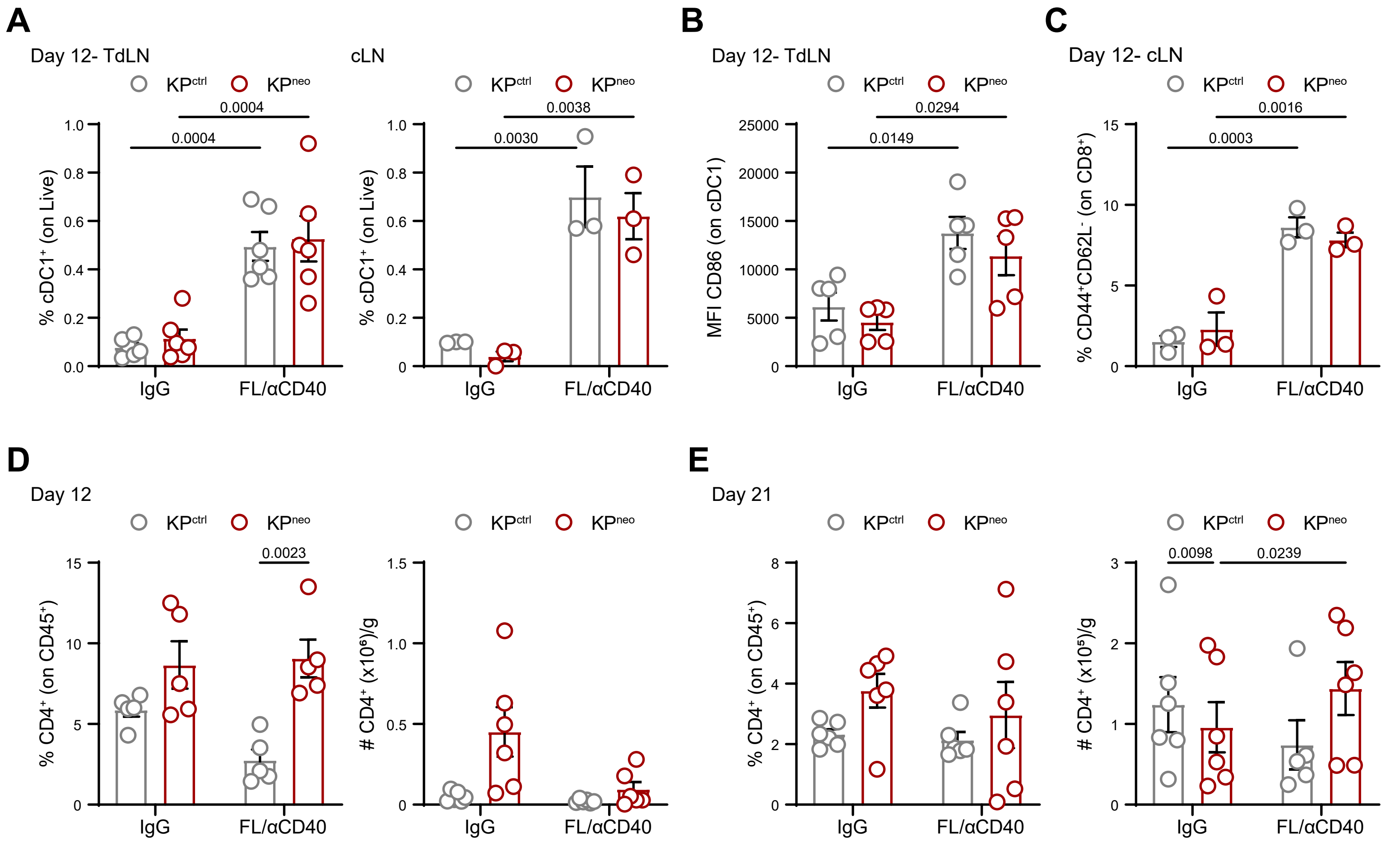

Supplementary Fig. 2

Supplementary Fig. 2| Immune profiling of DCs and CD4 T cells after DC-Therapy.

A) Frequency of cDC1 in the tumor-draining lymph node (tdLN) (left) and in the contralateral non-draining lymph node (cLN) (right) at day 12 post-tumor challenge in s.c. settings (n=3 left, n=6 right). **B)** CD86 expression on cDC1 in tdLN (n=5). **C)** Percentage of CD8 T cells expressing effector/effector memory markers ($CD44^+/CD62L^-$) in cLN at day 12 post-tumor challenge (n=3). **D,E)** Frequencies (left, n=5) and absolute numbers (right, n=8, for KP^{ctrl} FL/αCD40 n=6) of tumor-infiltrating CD4 T cells at day 12 (**D**) and 21 (**E**) (n=6). Two-way ANOVA followed by Tukey's post-test in **A-E**. All data are plotted as mean ± SEM and represent one out of two independent experiments. Source data are provided as a Source Data File.

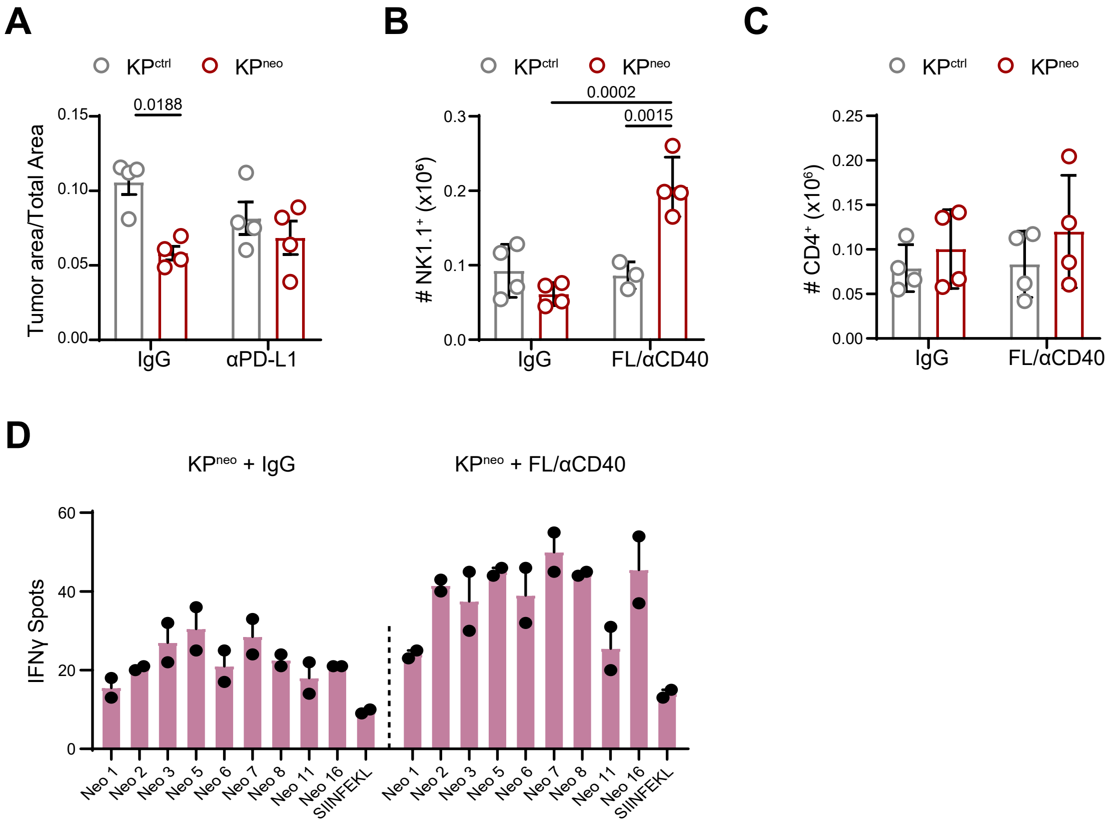

Supplementary Fig. 3

Supplementary Fig. 3| Profiling of DCs, NK cells and CD4 T cells in DC-therapy treated lungs in KP^{ctrl} and KP^{neo} orthotopic tumor.

A) KP^{ctrl} and KP^{neo} tumors were implanted orthotopically in the lung and treated i.p. with αPD-L1 or control isotype at day 5 and 7 post-injection. Quantification of tumor burden 9 days after tumor challenge. Tumor burden was calculated as the ratio between tumor nodules and total lung area (n=4). **B-C)** Absolute numbers of NK cells (**B**) and CD4^{+} T cells (**C**) in tumor-bearing lungs with KP^{ctrl} or KP^{neo} tumors upon FL/αCD40 or IgG, quantified by FC (n=4, one out of two independent experiments). **D)** IFN-γ ELISpot showing the specificities of CD8^{+} T cells isolated from KP^{neo}-bearing lungs under DC-therapy or IgG to unique peptides. Individual dots are technical replicates from one representative experiment (pooled CD8 T cells from 3 mice), out of four performed. Two-way ANOVA followed by Sidak's post-test in **A** or Tukey's post-test in **B**. All data are plotted as mean ± SEM. Source data are provided as a Source Data File.

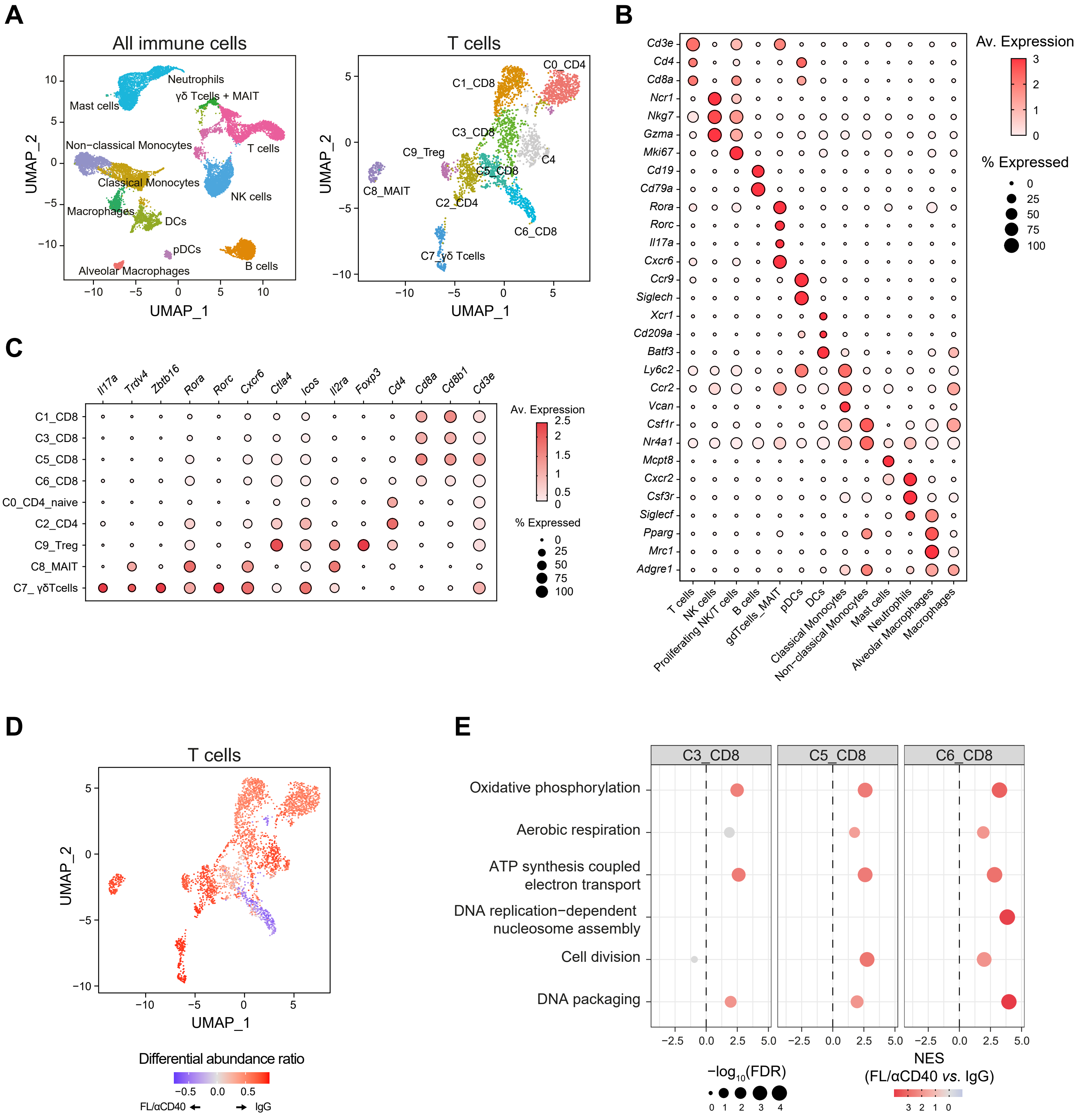

Supplementary Fig. 4

Supplementary Fig. 4| Remodeling of immune cells upon FL/αCD40 therapy.

A) CD45$^+$ cells were sorted from total lung tissues carrying KP^{neo} tumors treated with FL/αCD40 therapy or control IgG and analyzed by scRNA-seq. UMAP visualization of CD45$^+$ clusters (left), and T cells clusters (right), data are merged from therapy and control samples. **B,C)** Dot plot showing expression of selected genes (negative values set to zero) defining CD45$^+$ clusters **(B)** and all T cells subsets **(C).** **D)** UMAP visualization of merged scRNA-seq data showing cells in the T cell clusters colored by differential abundance ratio between experimental conditions (DA-seq algorithm). **E)** GSEA performed on expressed genes in different CD8 T cell clusters, ranked by log_2FC for FL/αCD40 versus IgG comparison, using biological processes gene ontologies as gene sets. Normalized enrichment scores (NES) and significance are reported for selected significant terms. **A-E)** scRNAseq data correspond to a pool of 4 animals per group.

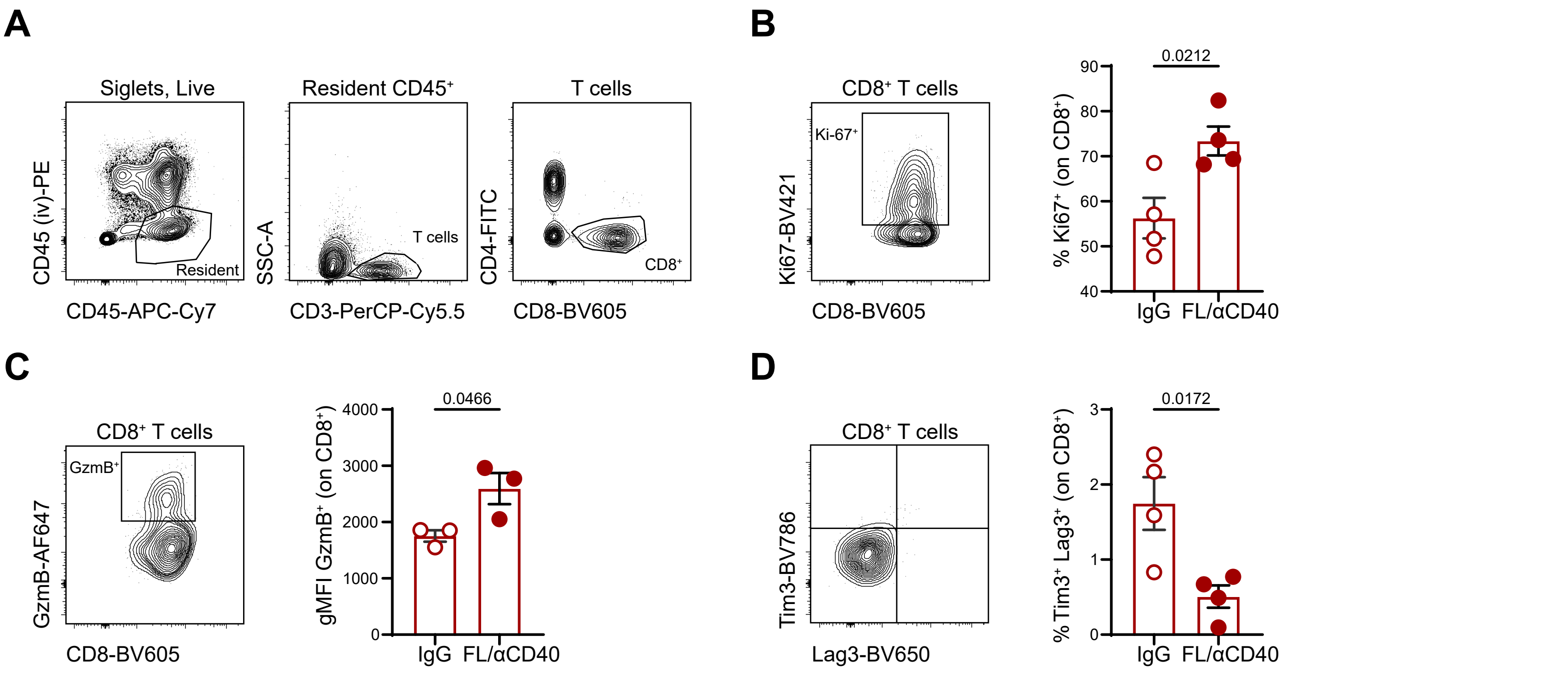

Supplementary Fig. 5

Supplementary Fig. 5| Remodeling of lung resident T cells upon FL/αCD40 therapy.

KP^{neo} tumor were implanted orthotopically in WT mice and treated with FL/αCD40 or control isotype. At the endpoint (day 9) mice mire injected intravenously with 3 µg of anti-CD45-PE to exclude circulating immune cells. **A)** Gating strategy showing the exclusion of circulating CD45^{+} cells and further gating to identify CD8^{+} T cells. **B)** Dot plot and quantification of fraction of resident Ki67^{+} CD8^{+} T cells (n=4). **C)** Dot plot and quantification of geometric MFI (gMFI) of GzmB in resident CD8^{+} T cells (n=3). **D)** Dot plot and quantification of fraction of exhausted Tim3^{+}Lag3^{+} CD8^{+} T cells (n=4). Two-tailed Student *t-test* in **B**-**D**. Data are plotted as mean ± SEM and represent one out of two independent experiments. Source data are provided as a Source Data File.

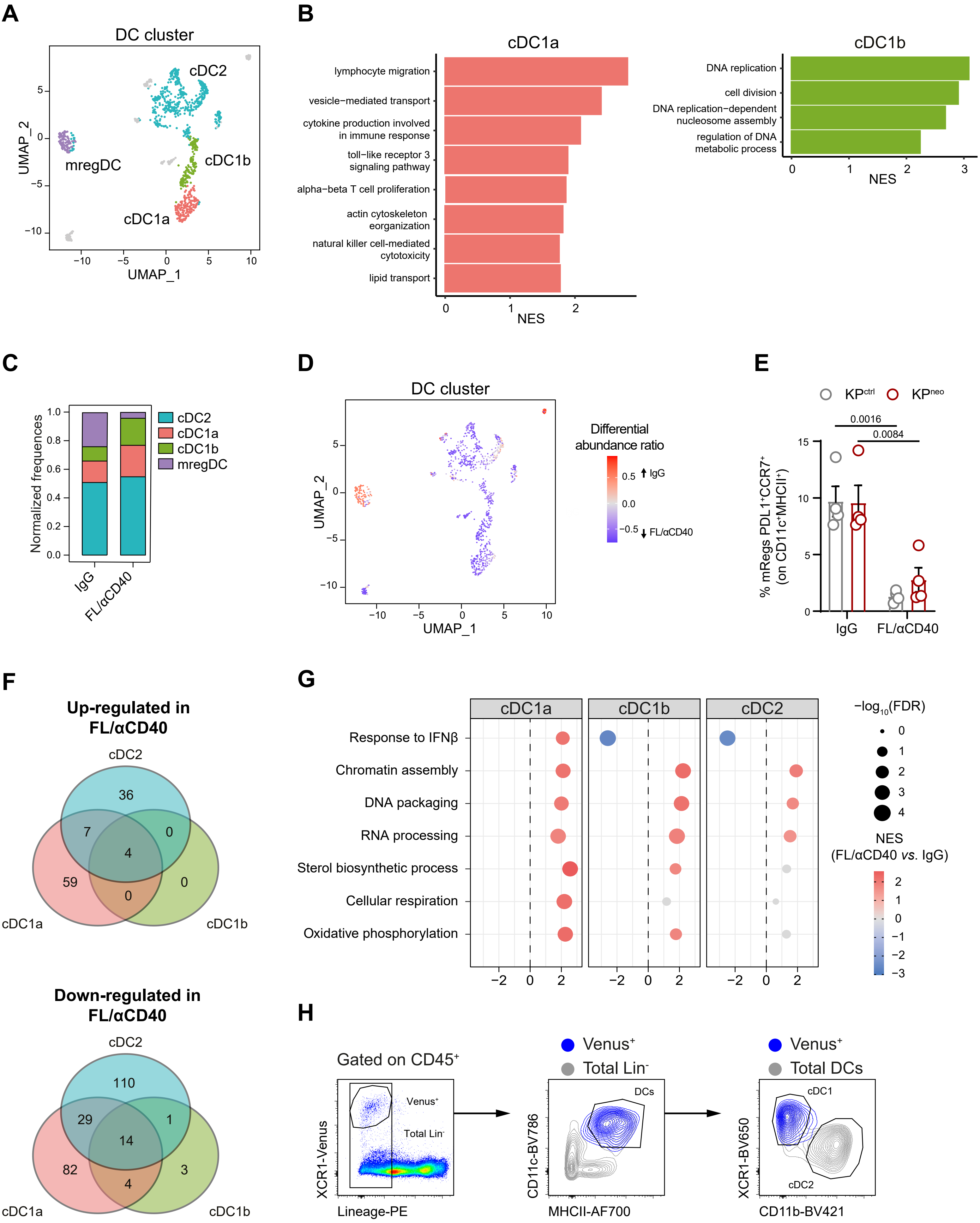

Supplementary Fig. 6

Supplementary Fig. 6| Remodeling of lung resident cDC upon FL/αCD40 therapy.

A) UMAP visualization of merged scRNA-seq data showing cells in the DCs cluster colored by sub-cluster (left), or experimental condition (right). **B)** Gene set enrichment analysis (GSEA) performed on expressed genes of cDC1s clusters ranked by log_2FC (cDC1a vs cDC1b comparison), using gene ontologies-biological processes as gene sets. Normalized enrichment scores (NES) are reported for selected significant terms. GSEA performed on genes ranked by log_2FC in cDC1a and cDC1b. **C)** Cluster composition per condition, in control (IgG) or therapy treated group (FL/αCD40). **D)** UMAP visualization of merged scRNA-seq data showing cells in the DC clusters colored by differential abundance ratio between experimental conditions (DA-seq algorithm). **E)** Frequencies of mregsDCs in KP^{ctrl} and KP^{neo} tumor bearing lungs in control and therapy treated groups. mregsDCs were identified as PD-L1^{+}CCR7^{+} on MHCII^{high} CD11c^{+} cells (n=4, one out of two independent experiments). Source data are provided as a Source Data File. **F)** Venn diagrams show the number of common and unique upregulated or downregulated DEGs in therapy-treated vs control samples in the 3 main cDCs subsets. **G)** GSEA performed on all genes in each of the 3 major DCs clusters (treated vs control). NES and significance are reported. **A-D,F,G)** scRNAseq data correspond to a pool of 4 animals per group. **H)** Gating strategy and representative dot plots to identify XCR1-Venus cDC1 in control and FL/αCD40 treated lung tissues. Lin (lineage) includes B220, CD3ε, CD19, F4/80, Ly6C, Ly6G and NK1.1. In gray is depicted the classical gating strategy used to identify cDC1 and in blue is overlayed the Venus^{+} populations, showing the unequivocally identification of cDC1. Two-way ANOVA followed by Sidak's post-test in **E**. Data are plotted as mean ± SEM and represent one out of two independent experiments.

Supplementary Table 1. List of neoAgs. Putative neoantigens identified in KP^{ctrl} and KP^{neo} cells are listed based on their predicted affinity (IC_{50}) for MHC-I and their expression levels (TPM). (*) was used to generate Dextramer for neoAgs-specific CD8 T cell identification by FC.

	KP^{ctrl}	KP^{neo}	Predicted IC_{50}	Expression
SNV/Mb	46.91	57.63		
Frameshifts	13	104		
Predicted neoAgs	184	273		
Expressed neoAgs				
Shared neoAgs				
Name				
Sh1*	Eif3h - F84I - LEITNCFPI	Eif3h - F84I - LEITNCFPI	468.28	325.75
Sh2	Zfp106 - A656T - STSPCNSTVL	Zfp106 - A656T - STSPCNSTVL	6.20	78.65
Sh3	Zfp106 - C659R - ASPRNSTVL	Zfp106 - C659R - ASPRNSTVL	21.77	78.65
Sh4	Zfp106 - I1257F - SVYPAFPAV	Zfp106 - I1257F - SVYPAFPAV	23.00	78.65
Sh5	Zfp106 - I1257N - SSVYPANPAVI	Zfp106 - I1257N - SSVYPANPAVI	133.39	78.65
Sh6	Sppl2a - A74P - LSLMNLTGTPL	Sppl2a - A74P - LSLMNLTGTPL	64.67	38.05
Sh7	Slc30a4 - G64R - VNRAHPAL	Slc30a4 - G64R - VNRAHPAL	125.28	36.85
Sh8	Aurkb - R44W - SALALVNWF	Aurkb - R44W - SALALVNWF	1162.21	35.97
Sh9	Glod4 - K14R - FKVRNRFQTV	Glod4 - K14R - FKVRNRFQTV	1521.89	35.97
Sh10	Rnf130 - V198L - LSISFIVL	Rnf130 - V198L - LSISFIVL	47.63	30.10
Sh11	Glod4 - K14E - FKVENRFQTV	Glod4 - K14E - FKVENRFQTV	1014.62	25.30
Sh12	Tmem87a - F145L - FSGDLTHRLPL	Tmem87a - F145L - FSGDLTHRLPL	1466.96	25.15
Sh13	Ubr3 - L1686F - SVFASCLGL	Ubr3 - L1686F - SVFASCLGL	167.72	16.60
Sh14	Lyrm5 - D28G - AGYFKRRL	Lyrm5 - D28G - AGYFKRRL	115.12	15.93
Sh15	Chst14 - I172V - AGVLNNVDV	Chst14 - I172V - AGVLNNVDV	1249.32	13.68
Sh16	Spg11 - L217W - WIYIFNTM	Spg11 - L217W - WIYIFNTM	23.17	13.42
Sh17	Ovca2 - T32A - KALRGRAEL	Ovca2 - T32A - KALRGRAEL	1466.52	10.67
Sh18	Nlgn2 - V210A - AAYGNVIVA	Nlgn2 - V210A - AAYGNVIVA	637.98	7.90
Sh19	Casc5 - R950S - SAVEINNETSL	Casc5 - R950S - SAVEINNETSL	262.32	6.17
Sh20	Ttc28 - S616R - AAPYYEQYLRL	Ttc28 - S616R - AAPYYEQYLRL	185.41	5.84
Sh21	Hebp1 - V86L - VSFALFPNE	Hebp1 - V86L - VSFALFPNE	146.75	3.83
Sh22	Naip2 - N540Y - LLYRFQLV	Naip2 - N540Y - LLYRFQLV	32.91	2.41

Unique neoAgs				
	Eprs - L428I - YSRLNINNTVL		363.17	120.81
Neo 1		Eif3l - I118F-FAPQVGNDAVF	1201.6	325.55
Neo 2		Itgb1 - G266V-IVWRNVTRL	65.25	304.91
Neo 3		Cers2 - Y142H-ASWRFTHYL	16.11	187.48
Neo 4		Lmo7 - L1273P-PMVLNSNSI	1522.28	130.28
Neo 5		Sept7 - F48L-RGLEFTLM	125.36	124.00
Neo 6		Cant1 - T392A-SSFKFIPNA	24.22	53.79
Neo 7		Uchl3 - T90S-QSISNACGTI	59.4	43.49
Neo 8		Nup205 - Frameshift *-VNNEFEKL	203.59	40.11
Neo 9		Mki67 - M681T-YKMLNNLTL	104.48	40.02
Neo 10		Ppp2r5a - K401R-IMFASLYRI	118.72	29.30
Neo 11		Arhgap5 - L1410S-SSICFWPTL	12.68	28.07
Neo 12		Ankrd17 - Y2324H-SGIVNMDTPH	1319.17	27.32
Neo 13		Skap2 - I86T-FAGPADTTSL	1059.24	22.48
Neo 14		Uhrf1bp1l - Frameshift* -VVVVYTEL	12.7	21.06
Neo 15		Utp15 - H248R-VSLKNHRKTV	1631.27	17.60
Neo 16		Foxj3 - T361A-AAGSNSVAQV	1652.93	15.65
Neo 17		Ecd - P59S-YIWQNQSFNL	225.69	15.24
Neo 18		Zfx - Frameshift *-VTLRLQIRL	125.27	13.92
Neo 19		Cep192 - N18S-SSLLGNSEVL	46.9	11.98
Neo 20		Rin3 - H589R-VSFASVFRAFL	17.59	10.24
Neo 21		Runx2 - V149A-VAFKAVAL	27.98	8.88
Neo 22		B4galt2 - A40P-QHLPFFSRF	128.6	7.68
Neo 23		Casc5 - R950S-SAVEINNETSL	262.32	7.60
Neo 24		Stxbp5 - M655L-FGNCNGIAL	46.27	7.30
Neo 25		Lig4 - Frameshift *-LAYRLVTL	36.8	6.86
Neo 26		Exph5 - Frameshift *-ISLRQLACFL	1212.89	1.75

Supplementary Table 2. FC antibodies. List of fluorescent conjugated primary anti-mouse antibodies used in flow cytometry.

Antibodies Flow cytometry		**Provider**	**Catalog number**	**Dilution**
**B220**	FITC	BioLegend	103206	1:200
**B220**	PE	BioLegend	103208	1:200
**CD11b**	BV421	BioLegend	101251	1:500
**CD11c**	BV786	BioLegend	117335	1:200
**CD19**	FITC	BioLegend	115505	1:200
**CD19**	PE	PharmaMingen	09655B	1:200
**CD3**	PercPCy5.5	BioLegend	100328	1:200
**CD3**	Alexa 700	BioLegend	100215	1:200
**CD3**	FITC	BioLegend	100305	1:200
**CD3**	PE	BioLegend	100206	1:200
**CD4**	BV785	BioLegend	100453	1:200
**CD4**	FITC	BioLegend	100405	1:200
**CD44**	PE	BioLegend	103007	1:200
**CD44**	FITC	BioLegend	103005	1:200
**CD45**	APC/Fire™ 750	BioLegend	103154	1:200
**CD45 (for IV)**	PE	BioLegend	103106	3 µg per mouse
**CD62L**	BV650	BioLegend	104453	1:400
**CD8**	APC	BioLegend	100712	1:200
**CD8**	BV605	BioLegend	100743	1:400
**CD86**	BV605	BioLegend	105037	1:400
**F4/80**	FITC	BioRad	MCA497A488T	1:200
**F4/80**	PE	eBioscience	12-4801-82	1:200
**GzmB**	Alexa fluor 647	BioLegend	515405	1:100
**IFNγ**	PE	BioLegend	505808	1:100
**IFNγ**	BV421	BioLegend	505829	1:100
**IL-12**	PE	BD	554479	1:100
**Ki-67**	Clone D3B5	Cell Signaling	9129S	1:400
**Ki-67**	BV421	Invitrogen	404-5698-80	1:400
**Ly6C**	FITC	Invitrogen	53-5932-82	1:200
**Ly6C**	PE	Invitrogen	53-5932-82	1:200
**Ly6G**	FITC	BioLegend	127605	1:200
**Ly6G**	PE	BioLegend	127608	1:200
**MHCI**	PE	BioLegend	116507	1:200
**MHCII**	Alexa fluor 700	BioLegend	107622	1:400
**NK1.1**	FITC	BioLegend	108705	1:200
**NK1.1**	Alexa fluor 700	BioLegend	156511	1:200
**NK1.1**	PE	BioLegend	108707	1:200
**PD1**	BV421	BioLegend	135221	1:100
**PD-L1**	efluor780	Invitrogen	46-5982-80	1:200
**TCF-1**	PE	Cell Signaling	14456S	1:100
**XCR1**	BV650	BioLegend	148220	1:200

Supplementary Table 3. Primers for RT-qPCR.

Primers RT-qPCR	***Sequence 5'-3'***	
**IFNγ Forward**	ATG AAC GCT ACA CAC TGC ATC	IDT
**IFNγ Reverse**	CCA TCC TTT TGC CAG TTC CTC	IDT
**Cxcr3 Forward**	GCCATGTACCTTGAGGTTAGTGA	IDT
**Cxcr3 Reverse**	ATCGTAGGGAGAGGTGCTGT	IDT
**GmzB Forward**	ACAAGGACCAGCTCTGTCCTT	IDT
**GmzB Reverse**	TGTCAGTTGGGTTGTCACAGC	IDT
**GusB Forward**	ACTGACACCTCCATGTATCCCAAG	IDT
**GusB Reverse**	CAGTAGGTCACCAGCCCGATG	IDT
**GAPDH Forward**	AGAAGGTGGTGAAGCAGGCAT	IDT
**GAPDH Reverse**	CGAAGGTGGAAGAGTGGGAGT	IDT

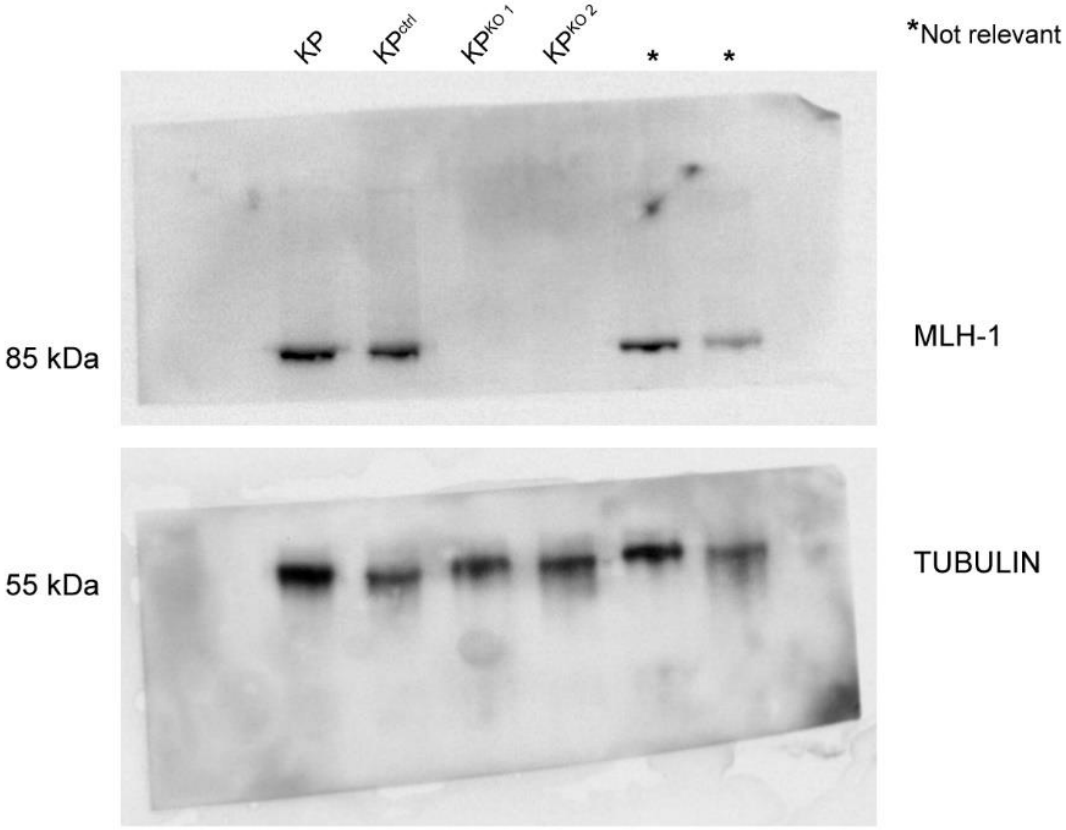

Uncropped WB membrane from Supplementary Fig. 1A.
