# Supplementary material for: Dendritic cell-targeted therapy expands CD8 T cell responses to bona-fide neoantigens in lung tumors
Source: Nat Commun. 2024 Mar 13;15:2280. doi: 10.1038/s41467-024-46685-y (PMC10937682; doi:10.1038/s41467-024-46685-y)
Supplement: Supplementary file 3 — Description of Additional Supplementary Files [file 41467_2024_46685_MOESM3_ESM.pdf]

Description of Additional Supplementary Files

Supplementary Data 1. All markers immune cells. This file contains all the genes, and their expression levels, used to define every cluster.

Supplementary Data 2. All markers T cells. This file contains all the genes, and their expression levels, used to define T cell clusters.

Supplementary Data 3. CD8_Cl3_Cl5_Cl6_DEGs_GSEA_post_vs_pre. This file contains all the differentially expressed genes and biological processes in CD8 T cell clusters.

Supplementary Data 4. CD4_Cl0_Cl2_DEGs_GSEA_post_vs_pre. This file contains all the differentially expressed genes and biological processes in CD4 T cell clusters.

Supplementary Data 5. All markers DCs_Cl1vsCl2_GSEA_BP. This file contains all the genes, and their expression levels, used to define dendritic cell clusters.

Supplementary Data 6. DCs_DEGs_GSEA_post_vs_pre. This file contains all the differentially expressed genes and biological processes in dendritic cell clusters.
