# Supplementary material for: Dendritic cell-targeted therapy expands CD8 T cell responses to bona-fide neoantigens in lung tumors
Source: Nat Commun. 2024 Mar 13;15:2280. doi: 10.1038/s41467-024-46685-y (PMC10937682; doi:10.1038/s41467-024-46685-y)
Supplement: Supplementary file 10 — Reporting Summary [file 41467_2024_46685_MOESM10_ESM.pdf]

nature portfolio

Corresponding author(s): Federica Benvenuti

Last updated by author(s): Feb 7, 2024

Reporting Summary

Nature Portfolio wishes to improve the reproducibility of the work that we publish. This form provides structure for consistency and transparency in reporting. For further information on Nature Portfolio policies, see our Editorial Policies and the Editorial Policy Checklist.

Statistics

For all statistical analyses, confirm that the following items are present in the figure legend, table legend, main text, or Methods section.

n/a	Confirmed	
[ ]	[x]	The exact sample size (*n*) for each experimental group/condition, given as a discrete number and unit of measurement
[ ]	[x]	A statement on whether measurements were taken from distinct samples or whether the same sample was measured repeatedly
[ ]	[x]	The statistical test(s) used AND whether they are one- or two-sided<br>*Only common tests should be described solely by name; describe more complex techniques in the Methods section.*
[x]	[ ]	A description of all covariates tested
[ ]	[x]	A description of any assumptions or corrections, such as tests of normality and adjustment for multiple comparisons
[ ]	[x]	A full description of the statistical parameters including central tendency (e.g. means) or other basic estimates (e.g. regression coefficient) AND variation (e.g. standard deviation) or associated estimates of uncertainty (e.g. confidence intervals)
[ ]	[x]	For null hypothesis testing, the test statistic (e.g. *F*, *t*, *r*) with confidence intervals, effect sizes, degrees of freedom and *P* value noted<br>*Give P values as exact values whenever suitable.*
[x]	[ ]	For Bayesian analysis, information on the choice of priors and Markov chain Monte Carlo settings
[x]	[ ]	For hierarchical and complex designs, identification of the appropriate level for tests and full reporting of outcomes
[x]	[ ]	Estimates of effect sizes (e.g. Cohen's *d*, Pearson's *r*), indicating how they were calculated

Our web collection on statistics for biologists contains articles on many of the points above.

Software and code

Policy information about availability of computer code

Data collection	BD FACSDiva Software (version 8.0.1).<br>ZEN Black Imaging Software (ZEISS) (version 14.0.27.201).
Data analysis	FlowJo V10 (version 10.8.2)<br>GraphPad (version 8.4.3)<br>Fiji (version 1.53c)<br>Ilastik (version 1.3.2)<br>R version (version 4.0.3)<br>Seurat package (version v 4.0.3)<br>scDblFinder R package (version v 1.4.0)<br>Seurat Wrapper package (version v 0.3.0)<br>Harmony algorithm (version v 0.1.0)<br>clusterProfiler (version v3.18.1)<br>fgsea package (version 1.26.0)

For manuscripts utilizing custom algorithms or software that are central to the research but not yet described in published literature, software must be made available to editors and reviewers. We strongly encourage code deposition in a community repository (e.g. GitHub). See the Nature Portfolio guidelines for submitting code & software for further information.

Data

Policy information about availability of data

All manuscripts must include a data availability statement. This statement should provide the following information, where applicable:

- Accession codes, unique identifiers, or web links for publicly available datasets
- A description of any restrictions on data availability
- For clinical datasets or third party data, please ensure that the statement adheres to our policy

Single-cell RNA-seq data have been deposited at ArrayExpress data repository under the accession number E-MTAB-12508. The TCGA publicly available data used in this study are available in the cBioPortal database https://www.cbioportal.org/study/summary?id=luad_tcga.
Whole exome sequencing data have been deposited at Sequence Read Archive (SRA) data repository under the accession number PRJNA1082088. RNA-seq data have been deposited at Gene Expression Omnibus (GEO) data repository under the accession de number GSE260743. All data generated in this study are provided in the Supplementary Information and Source Data file.

Research involving human participants, their data, or biological material

Policy information about studies with human participants or human data. See also policy information about sex, gender (identity/presentation), and sexual orientation and race, ethnicity and racism.

Reporting on sex and gender	N/A
Reporting on race, ethnicity, or other socially relevant groupings	N/A
Population characteristics	N/A
Recruitment	N/A
Ethics oversight	N/A

Note that full information on the approval of the study protocol must also be provided in the manuscript.

Field-specific reporting

Please select the one below that is the best fit for your research. If you are not sure, read the appropriate sections before making your selection.

[x] Life sciences [ ] Behavioural & social sciences [ ] Ecological, evolutionary & environmental sciences

For a reference copy of the document with all sections, see nature.com/documents/nr-reporting-summary-flat.pdf

Life sciences study design

All studies must disclose on these points even when the disclosure is negative.

Sample size	Sample size was determined based on previuos knowledge from our lab (PMID: 33854047, PMID: 35402081) and from other laboratories (PMID: 34714687), with a minimum of 3 animals per group. No statistical methods were applied a priori to calculate group sample size.
Data exclusions	All data were included unless there were technical issues with the experimental setup or data collection.
Replication	All experiments were performed independently at least twice. All replicates were successful.
Randomization	During this study, animals were randomized among experimental groups, and groups were allocated randomly in cages to avoid variability due to cage effects.
Blinding	Lung tumor area and CD8 numbers in lung nodules were automatically calculated by Ilastik using masks and batch analysis. A code number was asigned to each sample allowing us to be blind for further analysis. Flow cytometry and sequencing are quantitative measures and did not rely on qualitative assessments.

Reporting for specific materials, systems and methods

We require information from authors about some types of materials, experimental systems and methods used in many studies. Here, indicate whether each material, system or method listed is relevant to your study. If you are not sure if a list item applies to your research, read the appropriate section before selecting a response.

Materials & experimental systems

n/a	Involved in the study	
☐	☒	Antibodies
☐	☒	Eukaryotic cell lines
☒	☐	Palaeontology and archaeology
☐	☒	Animals and other organisms
☒	☐	Clinical data
☒	☐	Dual use research of concern
☒	☐	Plants

Methods

n/a	Involved in the study	
☒	☐	ChIP-seq
☐	☒	Flow cytometry
☒	☐	MRI-based neuroimaging

Antibodies

Antibodies used

(Antibodies-Fluorophore-Provider-Catalog number-Dilution)

Flow cytometry antibodies:

B220 FITC BioLegend 103206 1:200
B220 PE BioLegend 103208 1:200
CD11b BV421 BioLegend 101251 1:500
CD11c BV786 BioLegend 117335 1:200
CD19 FITC BioLegend 115505 1:200
CD19 PE PharmaMingen 09655B 1:200
CD3 PercPCy5.5 BioLegend 100328 1:200
CD3 Alexa 700 BioLegend 100215 1:200
CD3 FITC BioLegend 100305 1:200
CD3 PE BioLegend 100206 1:200
CD4 BV785 BioLegend 100453 1:200
CD4 FITC BioLegend 100405 1:200
CD44 PE BioLegend 103007 1:200
CD44 FITC BioLegend 103005 1:200
CD45 APC/Fire™ 750 BioLegend 103154 1:200
CD45 (for IV) PE BioLegend 103106 3 micrograms
CD62L BV650 BioLegend 104453 1:400
CD8 APC BioLegend 100712 1:200
CD8 BV605 BioLegend 100743 1:400
CD86 BV605 BioLegend 105037 1:400
F4/80 FITC BioRad MCA497A488T 1:200
F4/80 PE eBioscience 12-4801-82 1:200
GzmB Alexa fluor 647 BioLegend 515405 1:100
IFNγ PE BioLegend 505808 1:100
IFNγ BV421 BioLegend 505829 1:100
IL-12 PE BD 554479 1:100
Ki-67 Clone D3B5 Cell Signaling 9129S 1:400
Ki-67 BV421 Invitrogen 404-5698-80 1:400
Ly6C FITC Invitrogen 53-5932-82 1:200
Ly6C PE Invitrogen 53-5932-82 1:200
Ly6G FITC BioLegend 127605 1:200
Ly6G PE BioLegend 127608 1:200
MHCI (H-2Kb) PE BioLegend 116507 1:200
MHCII (I-A/I-E) Alexa fluor 700 BioLegend 107622 1:400
NK1.1 FITC BioLegend 108705 1:200
NK1.1 Alexa fluor 700 BioLegend 156511 1:200
NK1.1 PE BioLegend 108707 1:200
PD1 BV421 BioLegend 135221 1:100
PD-L1 efluor780 Invitrogen 46-5982-80 1:200
TCF-1 PE Cell Signaling 14456S 1:100
XCR1 BV650 BioLegend 148220 1:200
Chicken anti-rabbit IgG Alexa fluor 488 Invitrogen A21441 1:500
Goat anti-rat IgG (H+L) Alexa fluor 555 Invitrogen A21434 1:500

Western blot Antibodies:

anti-MLH1 (clone EPR3894) Abcam Ab92312 1:1000

	anti-Tubulin (clone 11H10) Cell signaling 8777 1:5000<br>Goat anti-mouse IgG HRP Invitrogen 31430 1:5000<br>Goat anti-rabbit IgG (H+L) HRP Invitrogen 987244 1:1000<br><br>IHC Antibodies:<br><br>anti-CD8 (clone 4SM15) Invitrogen 14-0808-82 1:800<br>Goat anti-rat IgG polymer reagent Vector MP-7404 pure<br><br>ELIspot and ELISA Antibodies:<br><br>Capture anti-IFNg (clone R4-6A2) BioLegend 505709 4 micrograms/mL<br>Detectio anti-IFNg (clone XMG1.2) BioLegend 505803 2 micrograms/mL<br><br>In vivo Antibodies:<br><br>anti-CD8 (clone YTS169.4) Bioxcell BP0117 200 micrograms<br>Control isotype IgG2a (clone 2A3) Bioxcell BP0089 200 micrograms<br>anti-PD-L1 (clone 10F.9G2) Bioxcell BE0101 200 micrograms<br>anti-CD40 (clone FGK4.5FGK45) Bioxcell BP0016-2 80 micrograms
Validation	Antibodies used for flow cytometry, IHC, ELISpot, ELISA and western blot are validated by the suppliers as indicated in the suppliers´ website. Datasheets are available in the suppliers' website using the catalogue number as a reference (provided above).<br>Blocking antibody as well as agonist antibody used for the in vivo experiments are validated by the suppliers as indicated in the suppliers' website.<br>Depletion of CD8 T cell in vivo using Anti-mouse α-CD8 Clone YTS169.4 Bioxcell Cat #BP0117 was validated by flow cytometry.

Eukaryotic cell lines

Policy information about cell lines and Sex and Gender in Research

Cell line source(s)	The KP cell line was isolated from primary lung tumors of C57BL/6 KP mice (K-rasLSLG12D/+; p53fl/fl mice)(Dimitrova et al., 2016) and kindly provided by Dr. Tyler Jacks. KPctrl and KP Mlh1 KO 1 and 2 (KPneo) cell lines were obtained in our laboratory using Crispr Cas9-based technology.
Authentication	The KPctrl, KP Mlh1 KO 1 and 2 cell lines were sequenced using whole exome sequencing.
Mycoplasma contamination	All cell lines were routinely tested for Mycoplasma and tested negative for Mycoplasma.
Commonly misidentified lines (See ICLAC register)	No commonly misidentified lines were used in the study.

Animals and other research organisms

Policy information about studies involving animals; ARRIVE guidelines recommended for reporting animal research, and Sex and Gender in Research

Laboratory animals	C57BL/6JOlaHsd were purchased from ENVIGO, Batf3-/- mice were kindly donated by Dr. Christian Lehmann (Erlangen University Hospital) and XCR1-Venus were kindly provided by Prof. Wolfgang Kastenmuller (Wurzburg Institute of System Immunology). OT-I (C57BL/6-Tg(TcraTcrb)1100Mjb/J) mice were purchased from Jackson Laboratories. All animales were used at 8-12 weeks old.<br><br>Housing conditions: C57BL/6JOlaHsd, Batf3-/-, OT-I and XCR1-Venus were maintained at the ICGEB animal Bioexperimentation facility in sterile isolators (12h/12h light/dark cycle, T 21°C ± 2°C, RH 55% ± 10%). Mice received a standard chow diet and water ad libitum.
Wild animals	No wild animals were used in this study.
Reporting on sex	During this study only females were used.
Field-collected samples	No field-collected samples are included.
Ethics oversight	Animal care and treatment were conducted with national and international laws and policies (European Economic Community Council Directive 86/609; OJL 358; December 12, 1987). All experiments were performed in accordance with the Federation of European Laboratory Animal Science Association (FELASA) institutional guidelines and the Italian law.

Note that full information on the approval of the study protocol must also be provided in the manuscript.

Plants

Seed stocks

N/A

Novel plant genotypes

N/A

Authentication

N/A

Flow Cytometry

Plots

Confirm that:

- [x] The axis labels state the marker and fluorochrome used (e.g. CD4-FITC).
- [ ] The axis scales are clearly visible. Include numbers along axes only for bottom left plot of group (a 'group' is an analysis of identical markers).
- [x] All plots are contour plots with outliers or pseudocolor plots.
- [x] A numerical value for number of cells or percentage (with statistics) is provided.

Methodology

Sample preparation

Tissues (lungs or tumor mass) were mechanically dissociated and digested using collagenase type-II (265 U/mL) and DNAse-I (250 U/mL) and incubated a 37°C for 30 minutes. Samples were filtered through a 100 µm filter to obtain a single cell suspension and red blood cells were lysed using ACK buffer (Biolegend). Lymph nodes (mediastinal or inguinal) were smashed and filtered through a 70 µm cell strainer.

Instrument

BD FACSCelesta
BD FACSAria II
BD LSRFortessa X-20

Software

BD FACSDiva Software version 8.0.1.
FlowJo V10 10.5.3

Cell population abundance

Cells were sorted using FACSAria II. Cell purity was confirmed by post-sort analysis and the cell were used when the purity was higher than 95% .

Gating strategy

Total cells were gated on FSC-A vs SSC-A plots as has been traditionally done. Single cells were gated on a SSC-A vs SSC-W plots, and dead cells were excluded using Acqua fluorescent reactive dye (Invitrogen). Gating strategies used to identify cDCs in tumor-bearing lungs are depicted within the paper, starting from live singlets. For cDC in tumor mass, cells were gated as CD45+, Lin(CD3, NK1.1, B220, CD19)-, CD11c+, MHCIIhigh, XCR1+, CD11b-, starting from live singlets. CD8 and CD4 T cells were gated as CD45+, CD3+, CD8+ or CD4+, starting from live singlets. NK cells were gated as CD45+, CD3- NK1.1+.Positive populations were defined using FMOs.

- [ ] Tick this box to confirm that a figure exemplifying the gating strategy is provided in the Supplementary Information.
